# People’s expectations and experiences of big data collection in the Saudi context

**DOI:** 10.7717/peerj-cs.926

**Published:** 2022-03-16

**Authors:** Muhammad Binsawad, Ghazanfar Ali Abbasi, Osama Sohaib

**Affiliations:** 1 Department of Computer Information Systems, King Abdulaziz University, Jeddah, Saudi Arabia; 2 Labuan Faculty of International Finance, Universiti Malaysia Sabah, Malaysia; 3 Faculty of Engineering and IT, University of Technology Sydney, Sydney, Australia

**Keywords:** Big Data, Privacy, Social computing, Emerging technologies

## Abstract

Big data and machine learning technologies facilitate various business intelligence activities for businesses. However, personal data collection can generate adverse effects on consumers. Big data collection can compromise people’s sense of autonomy, harming digital privacy, transparency and trust. This research investigates personal data collection, control, awareness, and privacy regulation on people’s autonomy in Saudi. This study used a hybrid analytical model that incorporates symmetrical and asymmetrical analysis *via* fuzzy set qualitative comparative analysis (fsQCA) to analyze consumer sense of autonomy regarding big data collection. The symmetrical shows that ‘Control’ had the most significant influence on people’s autonomy, followed by ‘Big data collection’ and ‘Awareness’. The fsQCA shows 84% of the variation, explaining the people’s autonomy.

## Introduction

The flow of information has significantly increased because of progression in web 2.0 technologies, which has given a massive quantum of data: ‘Big Data’ (Lichy, Kachour & Khvatova, 2017). Since the third parties are likely to gain access to the consumer’s private information, which is thought to be the prevalent concern associated with Big data (Helveston, 2016), there can be two situations wherein personal data is compromised. Firstly, when the private information is intentionally shared by the provider, which can entirely be confined (Crawford & Jason, 2014). In the second situation, the provider is unable to secure valuable information. Privacy contains multifaceted aspects. Hence, immeasurable damage is realized due to privacy invasions. Autonomy (AUTONOM) is a crucial aspect of privacy: a space where an individual can make decisions independent of others. Autonomy is “the ability of consumers to make autonomous and informed decisions without excessive power or undue influence” (Drumwright, 2018).

Many online consumers are ignorant that some entities are collecting their online web activities. For instance, when surfing the web or during online shopping, there are different levels, such as web browsers, affiliation companies, social network platforms, ad-serving agencies etc., where the consumers’ data is being acquired. Big data significantly focuses on consumer analytics (Erevelles, Fukawa & Swayne, 2016). Nevertheless, the experts must evaluate the impact of all these data and sources of information on consumers’ data control, for example, privacy (CPRC). The consumer should have full authority to maintain their data. Consumer trust needs to be established in the online setting.

The commercial perspectives of big data are generally emphasized in the existing literature (Erevelles, Fukawa & Swayne, 2016; Berntzen & Krumova, 2017). Nonetheless, we need to gain an insight into the concept of consumer autonomy being central to big data collection. Consumer behaviour is a promising area with forthcoming opportunities that the big data revolution will likely bring (Hofacker, Malthouse & Sultan, 2016). In the age of big data, the consumers’ perceptions of autonomy and choice need to be further investigated (André et al., 2018). Consumer purchasing decisions and behaviour are the benefits that businesses can realize owing to big data. Yet, the entire value potential of big data can be revealed by thoroughly understanding the dynamics of consumer autonomy.

In the digital day and age, the key challenges experienced by the consumers are this exchange between the benefits that personalization can offer and remaining autonomous decision-makers. The consumers are left with limited opportunities to make well-informed and deliberate choices, mainly when AI is likely to enhance the likelihoods of covert influence and manipulation. In terms of an individual scale and democracy on a large scale, consumer autonomy is vulnerable to possible threats (Grafanaki, 2016; Mik, 2016; Susser, Roessler & Nissenbaum, 2019).

The transactions in-store and online, social media, web browsing, and sensor-enabled devices are various sources to generate consumer data. Consumers provide their data by adhering to online terms and conditions at many places. Nonetheless, these consumers may be unaware of its intended use and that their data is being acquired. The consumer has not been granted permission for data collection purposes across various other instances. The privacy and integrity of the consumers might have been breached subject to their privacy boundaries.

As explained above, big data collection creates several ethical questions regarding consumers’ autonomy and their expectation of using their data. Businesses have various reasons and motivations to collect and use personal consumer data. Therefore, the primary aim of this study is to determine the multiple factors that can cause consumer autonomy concerns and how these concerns can be mitigated. Focusing on the Saudi context, the research question this study is investigating is, what factors impact people autonomy and control of their online data collection?

This structure of the paper is organized as follows: the following “Literature Review” review the prior research on big data, then the theoretical foundations are hypotheses development are discussed in “Theoretical Framework and Hypotheses Development”. “Research Methodology” presents the research methodology. “Results” presents the results, and finally, the study concludes with a discussion.

## Literature Review

Besides generating content through sensor networks or business deals, for example, purchase transactions and sales queries, another huge quantum of sources, such as mobile transactions, Internet clicks, social media and user-generated content, tend to generate the big data (George, Haas & Pentland, 2014). In addition, as quoted in Lichy, Kachour & Khvatova (2017), “the health care, genomics, operations management, engineering, finance and the industrial Internet have a significant role in the big data occurrence”. Nonetheless, there is a likelihood of disregarding consumers’ privacy concerns during big data acquisition. The actual privacy concerns relate to significance and ease of personalization (Aguirre et al., 2015). Researchers are highly focused on privacy in big data due to its relevance (Aguirre et al., 2015). Data can be collected through numerous sources and contexts, and the subject may not be informed of data acquisition. The growing number of resources, such as digital technologies, is an apparent reason for massive data generation. Digital technologies are progressing fast, resulting in more and more data. Hence, a desperate change has been noticed in an individual’s behaviour associated with internet use (Buttarelli, 2015).

E-commerce is rapidly advancing with its own merits and demerits. The maintenance of privacy, risks of taking consent and the adjustment of analytical marketing techniques are included among the disadvantages. It also entails products and services exclusion and price discrimination techniques. Besides offers for the consumers, the online market is uplifted by the growing progress in data acquisition, including buyer attitude and online browsing as well as the social network, significance of products and the history of credit.

Besides effectively utilizing the information for constructive analysis, the use of big data to identify the concealed information about the attitude of users is defined as big data consumer analytics (Hofacker, Malthouse & Sultan, 2016). Owing to technology, a usual consumer has emerged as an individual who keeps on generating structured, unstructured, and conventional data types. The dimensions above contribute to delineating big data and are referred to as three V’s, *i.e*. volume, velocity and variety (Lycett, 2013; Erevelles, Fukawa & Swayne, 2016). The speed with which big data is immensely developed *via* digital processing is velocity. The enormity feature is known as volume. New formats and data diversity is something known as variety. The images, words, videos and non-numeric forms are different data shapes, and hence the same cannot be conditional on usual statistical analysis. Several risks are associated with the commercial uses of big data. Security breaches and other allied risks can be experienced subject to massive personal data (Romanosky, Hoffman & Acquisti, 2014). Users are generally not likely to follow consent-based restraints if the data comes from various streams. When the data is meant to suggest pricing, sales and employment matters, a possibility is there that users will not wholly adhere to anti-discriminate laws (Helveston, 2016).

There are diverse aspects in the literature related to Big Data. For instance, privacy-related concerns (Boyd & Crawford, 2012), big data and its applications (Cerf, 2014), the consequences of the digital tools and technologies (Mantelero, 2014), the moral principles of collecting personal data (Bodrov & Ramzaev, 2015) in addition to financial trade-offs (Reed & Dongarra, 2015). In recent advancements, big data has played a role in accelerating economic growth. It has also yielded new ideas and efficiency enhancement. As a result, both providers and consumers realize the benefits (Helveston, 2016). A negative impact on the consumer’s health may be one of the shortcomings (Helveston, 2016).

The global economy is data-driven (Zhang, Yang & Appelbaum, 2015), enabling researchers to gain insight into the important information produced with every minute. The consumer behaviour choice should also be taken into consideration.

## Theoretical Framework and Hypotheses Development

Business ethics have been driven by the principles of social contract theory (Donaldson & Dunfee, 1994). Generally, this theory has three vital elements: harmony among moral agents, consent of the individual, and a method or device through which an agreement is obtained (Dunfee, Smith & Ross, 1999). Characterized by the beliefs of social contract theory, the integrative social contract theory was developed by Donaldson & Dunfee (1994), delineating the shared norms within the industry by adhering to which the organizations will be bound to behave ethically. Some organizations may have legal requirements to have privacy policies within some territorial boundaries. Besides improving the consumer’s trust, the information privacy concerns may also be curtailed because of the privacy disclosure policy of an organization (Culnan & Armstrong, 1999). Nevertheless, individuals must show consent (control) for the data collection and be aware of their data usage (awareness) so that the policies may be effective. Primarily, individuals might express their consent for the collection of their data; but they may be ignorant about control of their data for “re-use,” which leads to a perceived breach of privacy (Culnan, 1995).

As far as information boundary theory is concerned, Stanton (2003) emphasizes that the willingness of an individual to share the data would be determined by his/her observation about the organization that acquires the data, its expected usage, and any likely benefit of sharing the data (Stanton, 2003).

With the help of boundary “opening” and “closing” behaviours, individuals control the exposure of personal data (Stanton & Stam, 2003). Upon finding/realizing some benefit, an individual may be ready to share information (boundary opening). However, the data (boundary closing) can be withheld to mitigate risk.

Within an organization, an individual's trust can be influenced by Information privacy concerns (Malhotra, Kim & Agarwal, 2004). In the event of a trust deficit, the consumers may stop all future transactions with an organization if their information privacy concerns are beyond their patience. In line with informational boundary theory, consumers may exhibit boundary closing behaviours if the perceived risk is substantial. The study on e-commerce and trust has revealed that a mediating factor between the willingness to execute online transactions and information privacy concerns is none other than trust (Van Slyke et al., 2006).

**Figure 1:**
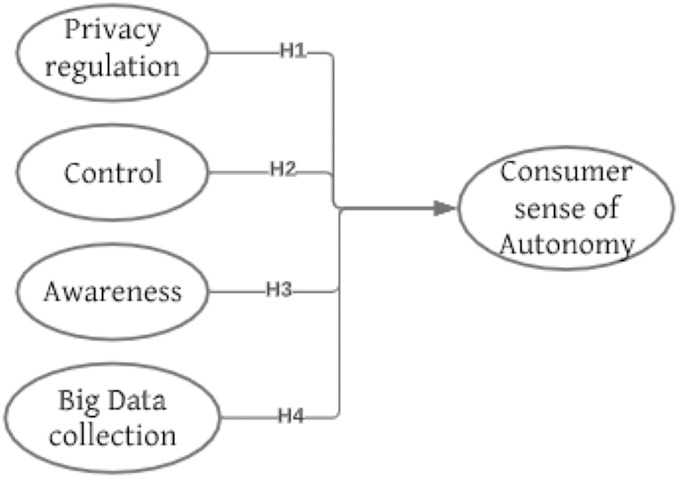
Research model.

Derived from the literature review, elements of awareness and control are common in the information boundary theory and the social contract theory concerning an individual's concept of information privacy. Figure 1 shows the research model.

### Privacy regulation (PRVREGU)

According to Vinuesa et al. (2020), the pace of technological development is one of the biggest challenges hampering sustainable AI development since individuals and governments do not appear to maintain this. Several key sustainability risks related to legislation and governance were indicated by Henriksson & Grunewald (2020), for instance: human rights violations (*e.g*., privacy, modern slavery), regulatory (*e.g*., failure to live up to the EU General Data Protection), diversity, unfairness, and inclusion (*e.g*., General Data Protection Regulation (GDPR)) besides non-adherence to ethical codes (*e.g*., responsible sales practice). Consumer welfare can be compromised owing to the lack of legislation regarding AI development. Nonetheless, in terms of consumers’ decision-making, privacy regulation is believed to be of extreme significance. This is attributed to Vinuesa et al. (2020), who claimed that “if not regulated properly, the consumer opinion towards a certain product or political cause might be influenced by the immense quantity of data generated by the people.”

According to Bleier, Goldfarb & Tucker (2020), the privacy regulation aims to regulate the extent to which consumers’ personal information can be tracked and used. For instance, the effectiveness of online advertising has reportedly been reduced by the privacy regulation (Goldfarb & Tucker, 2011), which can be important for consumer welfare because such a level of advertising is not looked-for. According to Pan (2016), consumer privacy is not threatened by acquiring and storing a single piece of quantitative data. However, it is instead the qualitative presumptions about a person’s qualities, such as their level of intelligence, personality traits, race, employee worth, and political identity, that are the focus. “When it is unclear which data is yielding which inferences, all obtained data might intrude on autonomy and disclose personal facts” (Pan, 2016). This data was obtained and used without the consumer’s knowledge or consent, and it was done in a shady manner. Since algorithms may intelligently identify unique patterns that humans cannot see, this compromises the customers’ autonomy.

Similarly, Grafanaki (2016) acknowledged that privacy is not confined to a single person; instead, it also holds “a public value in a manner that a democratic society and modernization both call for the individual autonomy and compromising it will bring damage to that society.” As far as online decision-making is concerned, the role of autonomy needs to be carefully examined concerning both the consumer and society.

Individual autonomy is significantly based on the level of privacy (Rossler, Glasgow & Glasgow, 2005; Lanzing, 2019). Therefore, regulation and legislation must be in place to protect privacy so that consumers’ autonomy could not be considerably affected, provided this course of action will dampen businesses’ opportunities to make use of data produced and examined about individual consumers. This has greater significance owing to the distinct mismatch among powerfully conveyed privacy concerns of consumers and their negligent actions about privacy.

According to Singla et al. (2017), consumers give high importance to the advantages realized from giving away personal data as compared to the privacy itself. It was also noticed that consumers would happily exchange information if they were likely to obtain personal benefits that may have a role in their consumer experience. The Cambridge Analytical case is a recent happening, due to which consumers have become cautious and anxious. Consequently, public awareness regarding collecting and using personal data has increased (Isaak & Hanna, 2018). Due to a lack of knowledge about constantly evolving AI data-driven technologies, it can be claimed that consumers are still oblivious of data privacy concerns. Therefore,

**H1:** Privacy regulation has a positive effect on consumer sense of autonomy. **Control and Awareness (CONT and AWARENES)**.

Researchers collect and store consumers’ data for analysis purposes. The information privacy concerns can be determined by various factors, such as privacy research, the awareness of personal data collection and its usage (Culnan, 1995, Clarke, 1999). The consumers’ ability to control their information also plays an essential role in affecting information privacy concerns (Clarke, 1999). With the acquisition of consumers’ records in big data, the information privacy concerns of the consumer will be affected by the two constructs of awareness and control (Bjørlo, Moen & Pasquine, 2021). Likewise, subject to the consumer’s control of their data and understanding, data usage can generate information privacy concerns. There are two sides to awareness; awareness will depict whether consumers are aware of their information acquisition and whether they are conscious of their data usage. Control also has various aspects (André et al., 2018). Consent for the provision of data and its legitimate usage are included in control related matters. The ability to withdraw consent or/and return the data is also included in the Control parameters. Moreover, the authority also restricts the re-use of data or secondary use of personal data. The information privacy concerns of an individual can be determined by the consumer awareness and control of the collection of data and its use.

**H2:** Control has a positive effect on consumer sense of autonomy.
**H3:** Awareness has a positive effect on consumer sense of autonomy.

### Big data collection (BDCLLC)

A fair data collection and use policy should incorporate a mechanism to notify consumers of the data collection and its intended use (awareness). For big data, the pseudonymization, anonymization and data masking tools have been thoroughly investigated by the researchers (Pan, 2016; Isaak & Hanna, 2018). For instance, they performed a field test on smartphones, not sending personal data to central servers. Thus, information privacy concerns get abridged (Sutanto et al., 2013). If significant control of data collection practices and data usage is enabled, the privacy concerns would be moderated. By following the big data disclosure policy, consumers will become aware of the procedures to withdraw or strip any of their identifiers (André et al., 2018).

Subject to the collection and data utilization without consumer permission, it should be ensured that the shared norms of data collection and use should be developed by organizations acquiring information from consumers or prospective consumers.

A collective standard among organizations will be needed given the potential competitive advantage of gathering and using big data. The authorities concerned should implement the legislative measures.

**H4:** Big data collection and use positively affect consumers’ sense of autonomy.

## Research Methodology

### Instrument development

This used a web-based survey instrument to collect data. The items were adapted from the previous studies (Lichy, Kachour & Khvatova, 2017; Helveston, 2016; Erevelles, Fukawa & Swayne, 2016, Nguyen & Solomon, 2019; Sohaib & Olszak, 2021). These scales underwent a process of adaptation to adapt to the particularities of our research and ensure content validity. Reflective indices were used to calculate all latent variables in this analysis. In terms of research instrumentations, we followed Hardy & Ford (2014), who indicated that sorting items generates contextual knowledge that aids in reducing questionnaire item misinterpretation. The sample was uniformly sized, the guidelines were italicized, and specific terms were outlined in bold to highlight essential themes to minimize respondent fatigue. We resolved linguistic misinterpretation by choosing simple, brief, and widely employed terms.

### Design and sample

The current study investigates what factors influence people’s autonomy to collect their personal data. An internet questionnaire has many advantages, including cost savings, time savings, and a broader reach, and is considered a much more suitable approach, particularly throughout a pandemic. Data were collected from Saudi citizens in King Abdulaziz University, Jeddah, Saudi Arab. The survey was conducted in English. The University of Technology Sydney granted Ethical approval to carry out the study (Ethical Application Ref: UTS HREC ETH16-0548). Information sheet consent was provided in the survey. Participation in the survey was voluntary, and no consent was required. However, information sheet was included in the survey which contains information about the purpose of the research and the nil/negligible risks associated while attempting the survey. Appendix A presents the survey questionnaire. A total of 249 available data were processed for statistical analysis out of 260 responses. Besides this, Kline (2013) speculated that an insufficient data set may harm the generalisability of research results. As a practice, this research used the inverse square root and gamma exponential technique to measure the minimum sample size, as suggested by Kock & Hadaya (2018). When using default values of 0.197 for minimal co-efficient and 0.05 for significant *p*-value stage, the required sample size for an inverse square root and gamma exponential strategies was determined to be somewhere between 198 and 214. As a result, considering the preceding debate, the sample size for this study (249) is entirely appropriate.

### Common method bias

CMV is inherent to behavioural studies, and it may occur primarily due to subjective judgment provided by the same respondents for predicting and targeting variables (Podsakoff et al., 2003). Hence, the researcher applies a mix of procedural and statistical remedies to counter the issues of CMV. Procedurally, respondents were assured that responses were anonymized to reduce respondents’ assessment apprehension. Furthermore, both the dependent and the independent variables were positioned in different portions of the questionnaire. Along with procedural remedies, researchers also applied several statistical techniques. Firstly, researchers used Harman’s single factor test since the unrotated single factor test explains 41.03% of all the variance.

Moreover, even though Harman’s single factor analysis is most commonly applied, researchers have demanded that it is not appropriate to engage with common method variance (Lowry & Gaskin, 2014). Therefore, following Bagozzi, Bergami & Leone (2003), researchers used a correlation matrix to validate it further. As per correlation matrix results, no value was found with more than 0.90 among the constructs, confirming that CMB was not the primary concern. Thereby, we conclude that this study did not encounter any CMV issues.

### Data analysis

Prior researchers have incorporated both symmetrical (SEM) and asymmetrical (fsQCA) econometric methodologies to gain a better understanding of complex human behaviour (Pappas & Woodside, 2021; Seyfi et al., 2021). For the significance of hypothesized relationships in the proposed model, researchers in this study have used PLS-SEM software. PLS-SEM has been adopted due to several reasons, such as it can test complex models (Ali Abbasi et al., 2022; Ashaari et al., 2021; Henseler, Ringle & Sarstedt, 2015; Chin, Marcolin & Newsted, 2003), does not involve normality, small sample size, and able to work without distributional assumptions (Abbasi et al., 2021). Along with symmetrical analytical tools (PLS-SEM) to explore the combinations of causal factors (causal complexities of four factors) leading to the outcome (people autonomy), asymmetrical modelling was performed by using fsQCA (Ragin, 2008). In fsQCA, Boolean algebra examines causal mechanisms to understand the proposed outcome (Ragin, 2008; Woodside, 2014). There are three steps involved using fsQCA, *i.e*., calibration, truth table and counterfactual analysis. Calibration converts the crisp values into fuzzy set values (Woodside, 2014; Seyfi et al., 2021). fsQCA treats each variable as if it were part of a loosely defined set whose membership values change over time (Ragin, 2008). FsQCA uses fuzzy set value systems to convert all survey data, such as “5” (strongly agree) to “1,” into fuzzy set value systems at the beginning of the process. Calibrating a set can be done in one of two ways: directly (identifying three distinct states of set membership: full non-membership, crossover, and full membership) or indirectly (evaluating the instances and rescaling the measurements) (Ragin, 2008). With empirical data, such as that from a Likert scale, it is recommended that the direct calibration method be utilized instead. Three points on the scale must be identified in this method. With the scale employed in this study, a direct calibration technique involving three anchors was adopted: respondents with a membership score of 0 were classed as complete non-members; 1 was used as the threshold for full membership, and 0.5 was used as the crossover point; (Woodside, 2014). It was then employed to turn all possible fuzzy set values into the threshold limit for each build using a non-linear stepwise logistics function incorporated into fsQCA software.

After the calibration process, the determination of the truth table is mandatory, allowing researchers to know several causal models, each containing a mix of several constructs to the desired outcome. With this technique, a set of appropriate circumstances may be generated, which are then referred to as configurations, causal models, solutions, or recipes, depending on their context (Pappas & Woodside, 2021). The researcher must first run the fuzzy set technique in the fsQCA software in order to construct a truth table with 2^k^ rows. Every possible combination of the k predictors is included in this strategy. It is used to determine the significance of the data using two parameters: consistency and coverage levels of 0.8 and 0.2. The coefficient of determination (R^2^) assesses the influence of every possible cause on a result, and it’s a good analogy to use while thinking about coverage. The greater the coverage value, the more thorough the coverage. The path coefficient (β), which measures the degree to which a causal condition contributes to the observed result, is comparable to consistency (Mehran & Olya, 2020; Ragin, 2008). Using the fsQCA programme, truth tables may be analyzed to find three types of solutions: difficult, moderate, and parsimonious. A subset of intricate solutions, intermediate solutions also contain simple answers, which the authors used in their research (Pappas & Woodside, 2021).

Lastly, counterfactual analysis, also known as necessary conditions analysis (NCA), enables scholars to determine the necessary and sufficient factors to predict the proposed outcome (Schneider & Wagemann, 2010; Coelho et al., 2021). In this regard, two criteria are used: consistency and coverage to select the excellent and necessary recipes (Seyfi et al., 2021). In NCA, a condition is essential if it has a consistency is more than 0.90, whereas it is found sufficient it has found to have coverage of more than 0.20.

## Results

249 observations were used for the analysis after removing incomplete or missing data. A total of 65% of participants were males, and females were 35%. A total of 55% of participants were 18–25 years old, followed by 35% were 26–34 years old. A total of 73% of participants spend more than 5 h per day using the Internet.

### Findings from symmetrical modelling (PLS-SEM)

#### Convergent validity

The average variance extracted (AVE) and factor loadings (λ) are used to determine convergent validity (Hair et al., 2019). As a rule of thumb, Hair et al. (2019) suggested a factor loading of ≥0.707 and a value of ≥0.50 for AVE is deemed acceptable for exploratory research. Findings in Table 1 illustrates that all the factor loadings and constructs AVE values are more than the threshold values. Thus, this study had no issue of convergent validity.

**Table 1:** Measurement model.

Construct	Items	Loadings	CR	AVE	VIF (AUTONOM)
Autonomy	Autonomy1	0.93	0.91	0.73	N/A
	Autonomy2	0.95			
	Autonomy3	0.92			
	Autonomy4	0.56			
Awareness	Awareness1	0.83	0.91	0.71	1.12
	Awareness2	0.92			
	Awareness3	0.87			
	Awareness4	0.75			
Big Data Collection	BDCollection1	0.80	0.84	0.57	1.66
	BDCollection2	0.77			
	BDCollection3	0.73			
	BDCollection4	0.73			
Control	Control1	0.95	0.99	0.95	1.55
	Control2	0.98			
	Control3	0.98			
	Control4	0.98			
Privacy Regulation	PrvRegulation2	0.73	0.83	0.62	1.02
	PrvRegulation3	0.79			
	PrvRegulation4	0.836			

#### Construct reliability

The composite reliability (CR) parameter was used to assess construct reliability inside this analysis. According to Hair et al. (2019), a construct has achieved satisfactory reliability when the CR is ≥0.70. Results in Table 1 illustrate that all the studied constructs have CR values above the threshold set by Hair et al. (2019).

#### Discriminant validity

Cross-loadings and Fornell and Larcker are two standards for evaluating discriminant validity. Each of these tests has recently been criticized, so Henseler, Ringle & Sarstedt (2015) proposed using hetrotrait-monotrait (HTMT) ratios to determine discriminant validity. When the constructs’ HTMT values are <0.85 or <0.90, a study has achieved discriminant validity, as per the HTMT criterion. The study achieved discriminant validity, as all of the HTMT values were less than the most restrictive threshold, *i.e*., 0.85, as seen in Table 2. As a result, the measurement model has been thoroughly validated.

**Table 2:** Discriminant validity (HTMT_0.85_).

	AUTONOM	AWARENES	BDCLLC	CONT	PRVREGU
AUTONOM					
AWARENES	0.52				
BDCLLC	0.60	0.39			
CONT	0.68	0.24	0.47		
PRVREGU	0.07	0.06	0.10	0.19	

#### PLS-SEM (structural model)

Once the study’s measurement model is established, analysis can proceed to the next stage of symmetrical analysis, *i.e*., examining the proposed hypotheses of the study. In this regard, the bootstrapping technique is applied in SmartPLS. Out of the four proposed hypotheses, H1(β = −0.151, *p* < 0.05) were found to have a negative effect on the proposed outcome. Thus, H1 was not supported whereas, awareness (β = 0.258, *p* < 0.05), big data (β = 0.256, *p* < 0.05) and control (β = 0.452, *p* < 0.05) were found to have a positive and significant effect on the proposed outcome of the study, therefore, H2, H3 and H4 were supported, *see* Table 3. Structural model examinations also measure the coefficient of determination (R^2^) which measures the variance explained by the exogenous constructs on the endogenous construct. Researchers in this study used PLS-SEM to examine the R^2^ value. Findings revealed that all the R^2^ values of the studied model are satisfactory as it surpasses the threshold suggested by Falk & Miller (1992), *i.e*., 0.10. Findings reveal that the R^2^ value for the proposed outcome was 56.8%. Stone-Geisser Q^2^ was also created to determine the predictive value of dependent constructs. The results also establish that the Q^2^ value was more than, *i.e*. (0.407), indicating the proposed model has established predictive relevance.

**Table 3:** Hypotheses testing.

Relationships	β	Mean	STDEV	T Stats	*P* Values	CI 5%	CI 95%	Decision
AWARENES → AUTONOM	0.258	0.262	0.049	5.328	0*	0.177	0.342	S
BDCLLC → AUTONOM	0.256	0.261	0.051	5.032	0*	0.184	0.347	S
CONT → AUTONOM	0.452	0.441	0.052	8.738	0*	0.351	0.525	S
PRVREGU → AUTONOM	−0.151	−0.133	0.071	2.129	0.017	−0.235	−0.004	NS

**Notes:**

β, path coefficient; CI, Confidence Interval; S, supported; NS, Not supported

* *p* value < 0.001; one-tail test.

#### Findings from asymmetrical modelling (fsQCA)

The fsQCA findings recommended good causal recipes to predict the desired outcome, *i.e*., intention to use remanufactured products (Table 4). Based on the estimation of the complex combination of eight predictors of behavioural preference towards remanufactured products, which are presented in Table 4, the fsQCA findings demonstrate that the configurations were adequate to forecast high and low scores of the survey’s outputs. Additionally, results from fsQCA generate three solutions. However, this study relies on the Boolean algorithm for intermediate solutions (see Table 4). Coverage and consistency are two criteria used in each intermediate solution with an acceptable threshold value of >0.20 and 0.80, representing the coefficient of determination and the correlation, respectively (Ragin, 2008). The overall solution consistency (0.99) value indicates how the five causal path parameters contribute to high consumer autonomy towards big data collection. Besides that, the overall solution coverage (0.84) expresses the probability that five causal recipes can predict a high level of people’s autonomy towards data collection.

**Table 4:** Truth table analysis.

High Autonomy			
Model: f = (CONT, BDCLLC, PRVREGU, AWARENES)			
FRQUENCEY CUTOFF	1		
CONNSISTENCY CUTOFF	0.945		
**Models**	**Raw Coverage**	**Unique Coverage**	**Consistency**
M1: ~CONT*BDCLLC*~PRVREGU	0.15	0.002	0.10
M2: CONT*PRVREGU*AWARENES	0.73	0.03	1
M3: ~CONT*~BDCLLC*PRVREGU*~AWARENES	0.10	0	0.95
M4: BDCLLC*~PRVREGU*AWARENES	0.27	0	0.10
M5: CONT*BDCLLC*AWARENES	0.80	0.05	1
Solution coverage:	0.84		
Solution consistency	0.99		
Low autonomy			
Model: f = (CONT, BDCLLC, PRVREGU, AWARENES)			
FRQUENCEY CUTOFF	1		
CONNSISTENCY CUTOFF	0.855		
**Models**	**Raw Coverage**	**Unique Coverage**	**Consistency**
M1: ~CONT*BDCLLC*~PRVREGU	0.61	0.026	0.85
M2: CONT*PRVREGU*AWARENES	0.63	0.042	0.87
Solution coverage:	0.662		
Solution consistency	0.823		

**Note:**

CONT, Control; BDCLLC, Big Data Collection; PRVREGU, Privacy Regulation.

Regarding causal recipes shown in fsQCA, it is observed that people autonomy can occur through the absence or presence of a given combination of variables. Findings in Table 4 reveal five causal recipes/models to predict the high desired outcome, *i.e*., high AUTONOM, the combination of the studied elements to generate a specific response as it is evident from Table 4 that consumers will have the higher outcome (AUTONOM) when they perceive higher BDCL and absence of CONT and PRVREGU (*see Model 1*). In comparison, Model 2 indicates that the presence of CONT, PRVREGU and AWARENES results in higher AUTONOM. Similarly, Model 3 shows that higher AUT is achieved when users perceive the presence of PRVREGU and the absence of CONT, BDCLLC and AWARENES. Whereas Model 4 depicts that the lack of PRVREGU and BDCLLC and AWARENES generates a higher proposed outcome. Lastly, model 5 indicates that the presence of CONT, BDCLLC and AWARENES will obtain higher AUTONOM.

#### Necessary condition analysis

Necessary conditions analysis entails determining if each antecedent condition’s personal effect is required for the recommended results. If a causal condition is always present when the result is established, it is said to be “necessary conditions.” If the consistency scores of a necessary component surpass 0.90, it can be verified (Schneider & Wagemann, 2010; Seyfi et al., 2021). The necessary conditions for generating higher behavioural intention to use remanufactured products were analyzed. Findings in Table 5 show that CONT and AWARENES are necessary conditions for achieving a higher level of autonomy towards data collection. This implies that without control and awareness, the consumer's autonomy towards data collection will not be achieved.

**Table 5:** Necessary condition analysis.

OUTCOME: AUTONOM	Consistency	Coverage
CONT	0.92	0.99
~CONT	0.19	0.91
BDLLC	0.89	0.98
~BDLLC	0.21	0.94
PRVREGU	0.81	0.96
~PRVREGU	0.30	0.98
AWARENES	0.91	0.97
~AWARENES	0.19	0.96

**Note:**

CONT, Control; BDCLLC, Big Data Collection; PRVREGU, Privacy Regulation; AUTONOM, Autonomy.

## Discussion and Conclusion

In terms of the symmetrical results of the model, CONT had the most significant influence on the people’s autonomy, followed by BDCLLC and AWARENES. On the other hand, the symmetrical findings show that PRVREGU had no impact on consumers’ autonomy. In addition, the results of fsQCA were compared to the six basic principles of complexity theory in this study (Pappas & Woodside, 2021). The five causal combinations generated 84% of the variation in the fsQCA results, explaining the people’s autonomy. The variance derived from the fsQCA study is more than the PLS-SEM model’s explanatory potential for AUTON, 56.8%.

Additionally, Model 5 (CONT, BDCLLC, and AWARENES) articulates the most precise explanatory potential for the study’s suggested outcome out of the five casual conditions. fsQCA further demonstrated that predicting AUTONOM based on just one condition or antecedent is insufficient, supporting Tenet 1. A complicated combination of CONT, BDCLLC, AWRENES, and PRVREGU is adequate for a consistently high score in the outcome condition, according to the second tenet of the recipe principle (Model 1 & Model 2, Table 4). As a consequence, Tenet 2 was also endorsed. The outcomes for tenet 3 revealed that five different models fit the desired outcome's conditions. Tenet 3 is thereby supported by the finding of alternate configurations.

Additionally, tenet 4: causal asymmetry states that a configuration for high outcome prediction isn’t always the inverse of the outcome negation recipe. According to the findings, the two low AUTONO causal models were not the polar opposites of the five high AUTONOM recipes. As a result, Tenet 4 was approved. Tenet 5 states that the features of other situations are used to evaluate each precedent’s perspective. As indicated in Table 5, CONT, AWARENES, PRVREGU, and BDCLLC played both positive and negative functions (see Table 4) in forecasting AUTONOM. As a result, Tenet 5 was approved. Tenet 6 states that each configuration should represent the views in some but not all cases and that each configuration’s coverage should be less than one. Table 5 shows that the coverage of each solution was less than 1, indicating that Tenet 6 was supported. Almost universally, six key principles of complexity theory have been validated by the proposed causal models. As a result, the use of complexity theory inveterates the intricate interactions of various aspects connected to CONT, PRVREGU, AWARENES, and BDCLLC in order to achieve the study's desired goal (autonomy).

The existing *corpus* of research is entirely based on symmetry modelling, namely structural equation modelling (SEM; Erevelles, Fukawa & Swayne, 2016; Berntzen & Krumova, 2017), primarily based on net effect modelling (Pappas & Woodside, 2021). Conversely, asymmetrical modelling is greatly limited in the big data collection and autonomy related to consumer decision making, and the absence of causally linked elements is noticeable. According to Douglas & Prentice (2019), the application of fsQCA elucidates phenomena that would be missed using symmetrical techniques. As a result, much as in real life, the data set’s constructions are rather intricate and not irreversibly well-adjusted (Pappas & Woodside, 2021). For instance, customer diversification in terms of control, privacy and regulation and data collection towards consumer autonomy may not be captured in a framework examined using conservative multivariate regression techniques. Thereby, due to the restrictions of previous existing papers, which used orthodox multivariate methods such as SEM to examine differences among countries, this study employs asymmetric affiliation between concepts and introduces the influence of a construct on the output, whilst complexity theory and configurational models necessitate that researched variables be necessary (Seyfi et al., 2021).

Moreover, depending on symmetric connections may be misleading because a single effect may well not impose in all cases, casting aspersions on the prospect of asymmetric correlation between the concepts (Woodside, 2014). For example, an elevated big data collection may be sufficient to contribute to high autonomy (AUTONOM). However, if control and privacy and regulations are missing, consumers could still have a favourable perception toward people’s autonomy. As a result, control may not be required for the conclusion. Therefore, requiring a different strategy acknowledges the linkages as asymmetric.

Thus, in addition to addressing the gap in the existing studies, researchers in this survey implemented a hybrid analytical model that incorporates symmetrical and asymmetrical analysis *via* fuzzy set qualitative comparative analysis (fsQCA) to analyze and discern the “sufficient” and “necessary” conditions for the several aspects that influence to a lower or higher degree of consumer autonomy, respectively (Seyfi et al., 2021). Therefore, the outcomes of fsQCA will help enhance prediction precision by revealing the causal models that might result in a more significant outcome. The fusion of fsQCA and symmetrical analysis (PLS-SEM) provides critical methodological weight to the argument by concentrating on consumer autonomy. People’s exposure to the data collection process and its use will reduce data privacy concerns and enhance awareness.

### Limitations and future work

This study has limitations like any other research study, which could be addressed in future studies. First, the data were collected in Saudi Arabia. Therefore, the generalizability of the findings is limited to one country. Second, the study didn’t consider all possible factors affecting online consumer autonomy. Future researchers could use various other variables, the moderating role of gender, *etc.*

## Supplemental Information

10.7717/peerj-cs.926/supp-1 Supplemental Information 1 Questionnaire.

10.7717/peerj-cs.926/supp-2 Supplemental Information 2 Raw data.

## Additional Information and Declarations

### Data Availability

The following information was supplied regarding data availability: The raw survey data are available in the Supplemental File.
