# Supplementary material for: People’s expectations and experiences of big data collection in the Saudi context
Source: PeerJ Comput Sci. 2022 Mar 16;8:e926. doi: 10.7717/peerj-cs.926 (PMC9044354; doi:10.7717/peerj-cs.926)
Supplement: Supplemental Information 1 [file peerj-cs-08-926-s001.docx]

**Appendix: Questionnaire**

**Control**

1. Consumer online privacy is a matter of consumers' right to exercise control and autonomy over decisions about how their information is collected, used, and shared.
2. Consumer control of personal information lies at the heart of data privacy.
3. I believe that online privacy is invaded when control is lost or reduced.
4. Companies should give me the control to opt-in/opt-out of the information they can collect about me.

**Awareness**

1. Companies seeking information online should disclose how the data are collected, processed, and used.
2. A reasonable consumer online privacy policy should have a clear and conspicuous disclosure.
3. I must be aware and knowledgeable about how my personal information will be used.
4. Companies who share my private information should inform me how they will share it.

**Big Data Collection:**

1. It usually bothers me when online companies ask me for personal information.
2. When online companies ask me for personal information, I sometimes think twice before providing it.
3. It bothers me to give personal information to so many online companies.
4. I'm concerned that online companies collect too much personal information about me.

**Privacy regulation**

1. Online companies should devote more time and effort to preventing unauthorised access to personal information.
2. Computer databases that contain personal information should be protected from unauthorised access—no matter how much it costs.
3. Data regulation and legislation must be in place to protect my data.
4. It is enough for me to know that the Saudi's company has a privacy policy disclosure, which means they will not misuse my information.

**Autonomy**

1. Online companies should not use personal information for any purpose unless it has been authorised by the individuals who provided input.
2. When people give personal information to an online company for some reason, the online company should never use the information for any other reason.
3. Online companies should never sell personal information in their computer databases to other companies.
4. Online companies should never share personal information with other companies unless authorised.
